# The association between conditioned pain modulation and psychological factors in people with chronic spinal pain: A systematic review

**DOI:** 10.1177/20494637241229970

**Published:** 2024-01-24

**Authors:** Michael Mansfield, Gianluca Roviello, Mick Thacker, Matthew Willett, Kirsty Bannister, Toby Smith

**Affiliations:** 1 School of Sport, Exercise and Rehabilitation Sciences, College of Life and Environmental Sciences, University of Birmingham, Birmingham, UK; 2 Centre of Precision Rehabilitation for Spinal Pain, University of Birmingham, Birmingham, UK; 3 Faculty of Medicine and Health Sciences, University of East Anglia, Norwich, UK; 4 Physiotherapy Department, Guy’s and St Thomas’ Hospitals NHS Foundation Trust, London, UK; 5 School of Physiotherapy, 655732 Royal College of Surgeons Ireland, Dublin, Ireland; 6 Central Modulation of Pain, Wolfson Centre of Age Related Diseases, Institute of Psychiatry, Psychology and Neuroscience, King’s College London, London, UK; 7 Warwick Medical School, 2707 University of Warwick, Coventry, UK

**Keywords:** Anxiety, catastrophising, conditioned pain modulation, depression, fear avoidance, spinal pain

## Abstract

Chronic spinal pain has negative effects on physical and mental well-being. Psychological factors can influence pain tolerance. However, whether these factors influence descending modulatory control mechanisms measured by conditioned pain modulation (CPM) in people with chronic spinal pain is unclear. This systematic review investigated the association between CPM response and psychological factors in people with chronic spinal pain. Published and unpublished literature databases were searched from inception to 23rd October 2023 included MEDLINE, EMBASE, CINAHL, and PubMed. Studies assessing the association between CPM response and psychological factors in people with chronic spinal pain were eligible. Data were pooled through meta-analysis. Methodological quality was assessed using the AXIS tool and the certainty of evidence measured through GRADE. From 2172 records, seven studies (*n* = 598) were eligible. Quality of included studies was moderate. There was very low certainty of evidence that depression (*r* = 0.01 [95% CI −0.10 to 0.12], I^2^ = 0%), and anxiety (*r* = −0.20 [95% CI −0.56 to 0.16], I^2^ = 84%), fear avoidance (*r* = −0.10 [95% CI −0.30 to 0.10], I^2^ = 70%) had no statistical associations with CPM responder status. Higher pain catastrophising was associated with CPM non-responder status (r = −0.19; 95% CI: −0.37 to −0.02; *n* = 545; I2: 76%) based on a very low certainty of evidence measured by GRADE. There is currently limited available evidence demonstrating an association between CPM response and psychological factors for people with chronic pain. Managing an individual’s chronic pain symptoms irrespective of comorbid psychological distress, should continue until evidence offer insights that more targeted interventions are needed.

## Introduction

Chronic spinal pain, including chronic neck pain (CNP) and chronic low back pain (cLBP), is one of the main causes of disability worldwide.^1^ It results in a dramatic socioeconomic burden.^1^ In 2019, worldwide, 568 million people suffered from low back pain (LBP), 223 million with CNP.^2,3^ Although anatomical causes can be identified in people suffering from spinal pain, this only account for 5%–10% of individuals.^4^ Instead, it is widely accepted that spinal pain has multi-dimensional interactions between biological, psychological and social factors.^1^ Regardless of whether there is a clear anatomical cause, or if the pain is defined as non-specific, psychological factors including catastrophising, anxiety, depression and kinesiophobia negatively influence pain intensity and disability.^1,3,5^ These manifestations may also impact on prognosis and transition from acute to chronic pain.^6,7^ For example, higher pain intensity and disability have been associated with catastrophising thoughts in people with cLBP and CNP.^6,8^

A systematic review reported that LBP and depressive symptoms might have reciprocal interactions.^9^ Moreover, depression and general anxiety have been identified as prognostic factors for pain chronicity in people with LBP.^7,10^ Similar findings supporting the impact of psychological factors were also observed in people with CNP.^11^ Given the ‘top-down’ influence associated with emotional processing on brainstem circuits that originate descending modulatory controls,^12^ a consideration of the role of psychological factors on spinal pain might reveal a connection between psychological and biological domains.^10^ Specifically, negative psychological factors and altered activity in endogenous modulatory pathways may share underpinning mechanisms and potential interactions.^13^

Activating an endogenous modulatory pathway is possible via the application of a conditioned pain modulation (CPM) protocol. Here, the inhibitory regulation that a conditioning stimulus has on the perception of a noxious test stimulus acts as a proxy measure of activity in the diffuse noxious inhibitory control pathway.^13,14^ Conditioned pain modulation protocols (‘pain inhibits pain phenomenon’) incorporate an assessment of an individual’s pain rating in response to a painful test stimulus (such as mechanical pressure) followed by a second pain rating assessment in the presence of a distally applied, painful conditioning stimulus. When the application of the conditioning stimulus concurrent to the test stimulus results in a decreased pain rating, the individual is said to have a functional descending inhibitory control.^13,14^ Specifically, during the CPM assessment, a participant rates their pain before and after conditioning. A CPM ‘responder’ profile correlates with a reduction in perceived pain on conditioning, while a CPM ‘non-responder’ profile correlates with no change, whilst a CPM ‘facilitator’ profile correlates with an increase in perceived pain on conditioning respectively. Several CPM protocols relying on different modalities have been adopted across the literature.^14^ Accordingly, participant CPM ‘responder’, ‘non-responder’ or ‘facilitator’ status must be interpreted carefully.

Crucially since the CPM protocol is applied in wakeful humans, psychological variables are likely to influence ‘responder’, vs ‘non-responder’ vs ‘facilitator’ status.^15,16^ Unravelling potential associations between mood disorders and CPM status in people with chronic spinal pain, where knowledge regarding the neurotransmitter mechanisms highlight an association between noradrenergic and serotonergic brainstem and spinal circuits, could exploit the neuroplasticity of modulatory pathways and lead to positive biological effects for people with chronic pain.

To the best of the authors’ knowledge, no systematic review has examined this association. Consequently, we aimed to address this by performing a systematic review to investigate the association between CPM and psychological factors in people with chronic spinal pain.

## Methods

This systematic review was reported in accordance with the PRISMA checklist.^17^

### Eligibility criteria

Studies were included if they met all the following criteria:

a) Adult (aged 18 years and above) with chronic spinal pain, defined as pain of at least a 3-month duration with CNP and/or cLBP, and with pain extending between the upper cervical spine and/or the inferior gluteal fold.
b) Studies investigating CPM paradigms which have evaluated a painful test stimulus followed by a second evaluation either at the same time as a distant, painful conditioning stimulus (parallel paradigm) or in series after the painful conditioning stimulus has been withdrawn (sequential paradigm).^14^
c) Studies assessing psychological factors or mental health symptoms including depression, anxiety, kinesiophobia, fear avoidance and pain catastrophising. Studies were included if they measured these factors through validated questionnaires and/or outcome measures (e.g. Pain Catastrophising Scale (Sullivan, 1995 #8063), Hospital Anxiety and Depression Scale^18^).
d) Studies reporting one or more coefficients of correlation between CPM and psychological factors (depression, anxiety, kinesiophobia, fear avoidance and/or pain catastrophising).
e) Published in English or Italian language and were either case-control, cross-sectional or cohort study design.

No restriction on publication date was applied.

Studies were excluded if they met any of the following criteria:

a) Animal or cadaveric studies
b) Commentaries, editorials, single case studies, reports or laboratory data, books or book chapters, letters, conference posters or proceedings or study protocols.
c) Studies that included participants with chronic spinal pain attributed to trauma (e.g. whiplash-associated disorder and fracture), motor neuron lesion, myelopathy, post-surgery, systemic pathology or metabolic diseases.

### Search strategy

MEDLINE (OVID interface), EMBASE (OVID interface), CINAHL (EBSCO interface) and PubMed were searched from inception to 10th June 2022. This was subsequently updated to 23rd October 2023. The search strategy was developed using MESH terms where possible. The terms included in the search strategy were linked using the Boolean terms AND/OR. The search strategies used for each database are reported in Supplementary File 1. The risk of publication bias was limited by searching the grey literature on the British National Bibliography for report literature, OpenGrey and dissertation abstracts. Finally, the reference lists of included studies and relevant reviews on CPM were also searched.

All citations and abstracts of retrieved studies were exported to EndNote V.20 (Clarivate Analytics, 2020). This was used for the screening process after duplicate removal. The screening process was conducted by one reviewer (GR) and consisted of two parts. First, the title and abstract were screened against the eligibility criteria. Then, full-text records were obtained for potentially eligible studies and were screened by the same reviewer (GR). A second reviewer (MM) independently verified the decisions made at full-text stage. The PRISMA flow diagram was used to summarise the selection process.

### Data collection process and data items

Data from included studies were extracted by one reviewer (GR) and verified by a second reviewer (MM). Any discrepancies were resolved through discussion. Five domains were considered during the data extraction: the aim of the study, sample characteristics, CPM paradigm, psychological factors and measures for correlation analyses.

The extracted characteristics of the investigated population included: the type of spinal pain MSK disorder and its duration, age, pain intensity, disability level and gender. For the CPM paradigm, information on the applied protocol, which includes the conditioned stimulus and test stimulus, was extracted. Psychological factors or mental health symptoms were extracted with the questionnaire and/or outcome measure assessing this factor.

Data on the correlation coefficient between CPM response and psychological factors were extracted. Pearson and Spearman correlation coefficients were extracted based on what was reported in the included studies. When a correlation coefficient was not reported, it was computed from other information, such as the standardised beta coefficient, used when findings were written using a linear regression model. Otherwise, if the study population was divided into subgroups (e.g. CPM responder or facilitator status or based on psychological factors), the correlation coefficient was obtained by using the mean standardised difference between subgroups.^19^ To facilitate the interpretation of findings and ensure consistency across studies, the sign of the correlation was adjusted always to have a negative correlation to indicate the association between CPM response (obtained by CPM responder status) and positive psychological factors (e.g. lower depression/fear/pain catastrophising).

### Critical appraisal and risk of bias assessment

Two reviewers (GR and GN) independently assessed the methodological quality of the included studies. When necessary, disagreement was resolved by discussion. Critical appraisal was conducted using the Appraisal tool for Cross-Sectional Studies (AXIS tool), which mainly focuses on the quality of methods and results.^20^ The AXIS tool comprises of 20 items, including seven questions related to the quality of reporting, seven related to study design, and six to potential biases introduced in the study. Finally, the overall methodological quality of included studies is reported using the AXIS score ranging from 0 to 20, where higher scores represent greater quality.

### Data synthesis and meta-analysis

All included studies were assessed by one reviewer (MM) from a clinical perspective (e.g. diagnosis and variability in population characteristics) and study methodology in determining whether studies could be pooled together for synthesis. Sufficient clinical homogeneity was present with the included studies population, study design and CPM paradigm. This was discussed and agreed with a second reviewer (TS). If two or more studies reported data on a particular factor, a meta-analysis was conducted for each individual psychological factor to evaluate the association between CPM response and psychological factors. Before performing the meta-analysis, Fisher’s Z transformation was used to transform the correlation coefficients.^21^ An inverse Fisher’s Z transformation was then applied to obtain the pooled correlation coefficient of the meta-analysis. A random-effects model was used to conduct the meta-analyses because the CPM paradigms and the outcome measures of the psychological factors of interest had some variance between the studies.^22^

Statistical heterogeneity was assessed using I^2^ statistics, and different cut-off values were considered to describe the level of statistical heterogeneity. Specifically, statistical heterogeneity was reported as moderate, substantial, and extensive if the I^2^ statistics were between 40% and 60%, between 60 and 80%, or >80%, respectively.^22^

Grading of Recommendations, Assessment, Development and Evaluation (GRADE) was adopted to facilitate understanding outcomes quality and transparent grading of certainty in the included studies. GRADE has five domains assessing the certainty of evidence: Risk of bias; Imprecision; Inconsistency; Indirectness; Publication bias. One reviewer (MM) independently determined whether outcomes were very low, low, moderate or high certainty based on GRADE. This was verified by a second reviewer (TS).

## Results

**Figure 1.**
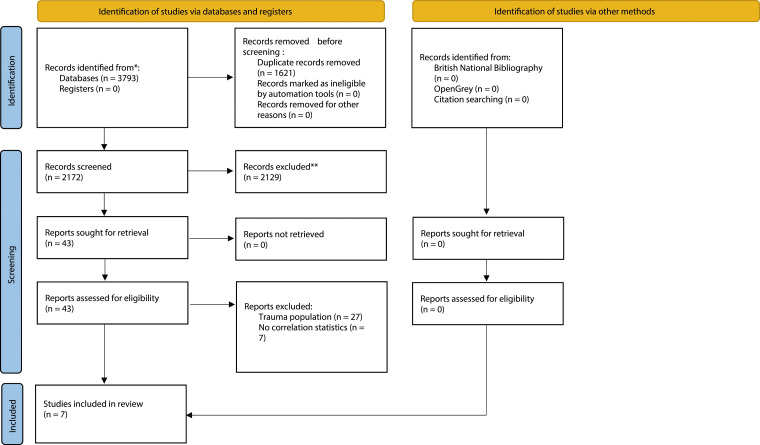
PRISMA flow diagram of included studies.

A summary of search strategy results is illustrated in Figure 1. In total, 3793 records were retrieved. The title-abstract screening was conducted for 2172 records which were obtained after the removal of duplicates. The inclusion of studies in the present review was completed after the full-text screening of 43 records. Finally, seven studies met the eligibility criteria and were included in the meta-analyses.^5,10,23–27^

### Characteristics of included studies

The characteristics of the included studies are reported in Table 1. All seven included studies assessed the relationship between CPM and psychological factors in people with cLBP.^5,10,23–27^ One study also recruited people with CNP.^23^ Overall, 598 people with spinal pain were analysed; 57% were females, with mean ages ranging from 37 to 60 years.

**Table 1.** Characteristics of included studies.

Study	Country, population, sample size (*N*)	Sex (F/M)	Age (Years)	Pain	Disability	CPM paradigm (Test/Conditioning stimulus)	Psychological factors	
							Outcome domain	Outcome measure
Christensen 2020	Denmark, cLBP, 22	12/10	41 ± 12	NRS: 6 [5–7]	RMDQ: 59.6 ± 18.2	Pressure: low back/Cold: right hand	Pain catastrophising	PCS
Dubois 2016	Canada, cLBP, 100	46/54	37 ± 12	VAS: 22.6 ± 16.3	RMDQ: 2.8 ± 2.6	Heat: low back/Cold: left hand	Pain catastrophising Fear avoidance	PCS FABQ
Gerhardt 2017	Germany, cLBP, 53	27/26	60 ± 11	NRS: 4.1 ± 1.9		Pressure: low back/Heat: thenar	Anxiety Depression	HADS
Martel 2013	United States of America, cLBP, 55	35/20	49 ± 10	NRS: 5.8 ± 1.9		Pressure: trapezius/Cold: contralateral hand	Pain catastrophising Anxiety Depression	PCS PASS BDI-II
Owens 2016	United States of America, cLBP, 25	13/12	58 ± 11	NRS: 6.0 ± 2.5	ODI: 52%	Pressure: forearm & trapezius/Cold: hand	Pain catastrophising	CSQ-R
Rabey 2021	Australia, cLBP, 273	163/110	50 [37–60]	NRS: 5.8 ± 1.9	RMDQ: 9 (6–13)	Pressure: low back/Heat: dorsum of the wrist	Anxiety and depression Fear avoidance Pain catastrophising	DASS-21 FABQ PCS
Vaegter 2017	Denmark, cLBP and CNP, 70	43/27	48 [20–86]	NRS: 6.7 ± 1.9	PDI: 37.2 ± 8.1	Pressure: right leg/Pressure: left leg	Pain catastrophising Kinesiophobia	PCS TSK

BDI-II: Beck Depression Inventory (45); cLBP: chronic low back pain; CNP: chronic Neck Pain; DASS-21: Depression Anxiety Stress Scale (36); FABQ: Fear-Avoidance Beliefs Questionnaire (50); HADS: Hospital Anxiety and Depression Scale (47); PASS: Pain Anxiety Symptoms Scale (26); PCS: Pain Catastrophising Scale (29); TSK: Tampa Scale for Kinesiophobia (23).

Seven studies used a test stimulus of pressure pain.^5,10,23–27^ The conditioned stimuli were reproduced using cold and heat stimuli in four^5,24,25,27^ and two studies,^10,26^ respectively. Pressure pain was also used as a conditioned stimulus in one study.^23^

The domains of the psychological factors assessed were pain catastrophising,^5,10,24,25,27^ kinesiophobia,^23,27^ anxiety,^10,26^ depression^10,25,26^ and fear avoidance.^10,27^

### Critical appraisal of included studies and certainty of evidence

The AXIS tool, scores ranged between 10 and 14 points out of 20 (Table 2). The recurrent methodological and reporting weaknesses were related to the CPM protocol and the presentation of findings. Specifically, the information on the level of expertise of people applying the CPM protocol was often missing (*n* = 4), as well as the basis of the choice in the protocol used to test CPM (*n* = 3). Moreover, few studies adopted the original English version of the questionnaires (*N* = 3). This may affect the validity of assessing the psychological domain of interest. The overall strength of evidence measured through GRADE is reported in Table 3. Across all psychological outcomes (Pain catastrophising, depression, anxiety and fear avoidance) the evidence demonstrated a very low level of certainty.

**Table 2.** The methodological quality of included studies was assessed using the AXIS tool.

Study	Intro	Methods										Results					Discussion		Other		AXIS score
	Q.1	Q.2	Q.3	Q.4	Q.5	Q.6	Q.7	Q.8	Q.9	Q.10	Q.11	Q.12	Q.13*	Q.14	Q.15	Q.16	Q.17	Q.18	Q.19^a^	Q.20	
Christensen, 2020	1	1	0	1	1	1	NC	0	0	1	1	1	0	NC	1	1	1	1	0	1	13/20
Dubois, 2016	0	0	0	1	0	0	NC	0	1	0	1	1	1	0	1	1	1	1	0	1	10/20
Gerhardt, 2017	0	1	0	0	1	1	NC	1	1	1	1	0	0	NC	1	1	0	1	0	1	13/20
Martel, 2013	1	1	0	0	1	1	NC	1	1	0	1	0	0	NC	1	0	1	1	0	1	13/20
Owens, 2016	1	1	0	1	1	1	1	1	1	0	0	0	1	1	1	1	1	1	0	1	12/20
Rabey, 2021	1	1	0	1	1	1	NC	1	0	0	1	0	0	0	0	1	1	1	0	1	12/20
Vaegter, 2017	1	1	0	0	1	1	NC	1	0	1	1	0	0	0	1	1	1	1	0	1	14/20

Key: 1 = ‘Yes’, 0 = ‘No’, NC = ‘Not clear/reported.’

Q.1 - Were the aims/objectives of the study clear?

Q.2 - Was the study design appropriate for the stated aim(s)?

Q.3 - Was the sample size justified?

Q.4 - Was the target/reference population clearly defined?

Q.5 - Was the sample frame taken from an appropriate population base so that it closely represented the target/reference population under investigation?

Q.6 - Was the selection process likely to select subjects/participants that were representative of the target/reference population under investigation?

Q.7 - Were measures undertaken to address and categorise non-responders?

Q.8 - Were the psychological factors and CPM response measured appropriate to the aims of the study?

Q.9 - Were the psychological factors and CPM response measured correctly using instruments that had been trialled, piloted or published previously?

Q.10 - Is it clear what was used to determined statistical significance and/or precision estimates?

Q.11 - Were the methods (including statistical methods) sufficiently described to enable them to be repeated?

Q.12 - Were the basic data adequately described?

Q.13 - Does the response rate raise concerns about non-response bias?

Q.14 - If appropriate, was information about non-responders described?

Q.15 - Were the results internally consistent?

Q.16 - Were the results for the analyses described in the methods, presented?

Q.17 - Were the authors’ discussions and conclusions justified by the results?

Q.18 - Were the limitations of the study discussed?

Q.19 - Were there any funding sources or conflicts of interest that may affect the authors’ interpretation of the results?*

Q.20 - Was ethical approval or consent of participants attained?

^a^Item is reverse scored (i.e. 0 is a positive, counts as a point)

**Table 3.** Certainty of evidence.

Study design	Study	Number of studies/patients	Risk of bias	Imprecision	Inconsistency	Indirectness	Overall strength of evidence
Observational chronic low back pain							
Pain catastrophising	(5) (27) (25) (10, 23, 24)	6/545	High	Serious	High	No seriousness	Very low
Anxiety	(10, 25, 26)	3/381	High	Serious	High	No seriousness	Very low
Depression	(10, 26)	2/326	High	Serious	High	No seriousness	Very low
Fear avoidance	(10, 27)	2/373	High	Serious	High	No seriousness	Very low
Observational chronic neck pain							
Pain catastrophising	(23)	1/70	High	Serious	Moderate	No seriousness	Very low

GRADE approach for psychological factors.

### Data synthesis and meta-analyses

#### Pain catastrophising

**Figure 2.**
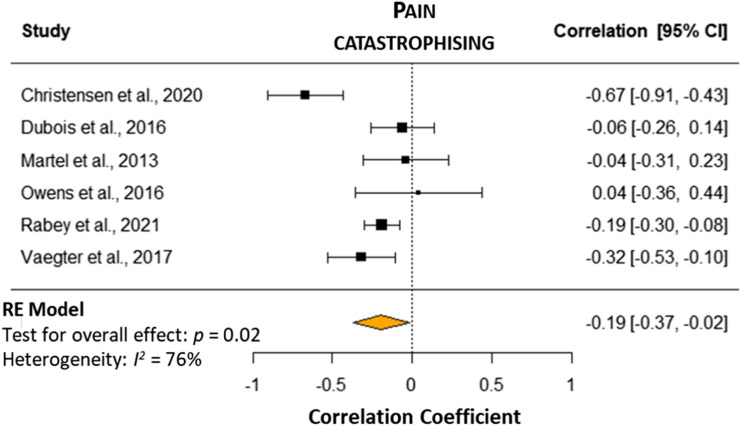
Forest plot with meta-analysis of pain catastrophising and CPM response in people with chronic spinal pain. A negative correlation indicates a relationship between higher pain catastrophising and lower CPM response.

There was a very low certainty of evidence from six studies (*n* = 545)^5,10,23–25,27^ (Table 3) detailing a weak correlation between pain catastrophising and CPM response (*r* = 0.19 [95% CI −0.37 to −0.02], *p* = .02, I^2^ = 76%; Figure 2). Therefore, higher pain catastrophising was associated with non-responder CPM status. Three studies reported no correlation when a cold stimulus was the conditioning stimulus ((r = −0.06 (95% CI −0.26 to 0.14),^27^ r = −0.04 (95% CI −0.31 to 0.23)^25^ and r = 0.04 (95% CI −0.36 to 0.44)^24^). The other three studies used pressure pain^23^, heat pain^10^ and cold pain^5^ as the conditioning stimulus. There was very low certainty evidence of a significant correlation when the test stimulus was applied to the low back region (r = −0.67 (95% CI −0.91 to −0.43),^5^ r = −0.19 (95% CI −0.30 to −0.08)^10^) and right leg using pressure pain (r = −0.32 95% CI −0.53 to −0.10).^23^ The other three studies targeted the low back region^27^ and trapezius.^24,25^

#### Anxiety

**Figure 3.**
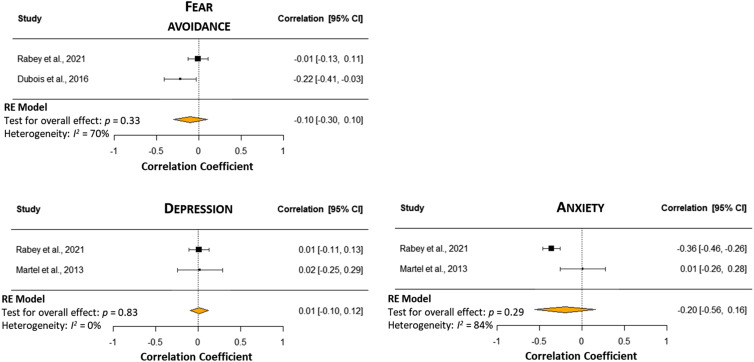
Forest plots with meta-analyses of different psychological factors and CPM response in people with chronic spinal pain. A negative correlation indicates an assocition between higher value of fear avoidance/anxiety/depression and lower CPM response.

There was a very low certainty of evidence and no correlation was present between anxiety and CPM response (*r* = −0.20 [95% CI −0.56 to 0.16], I^2^ = 84%; *n* = 328)^10,25^ (Figure 3).

#### Depression

There was a very low certainty of evidence, and no correlation was found between depression and CPM response (*r* = 0.01 [95% CI −0.10 to 0.12], I^2^ = 0%; *n* = 326)^10,25^ (Figure 3).

#### Fear avoidance

Two studies were pooled in the meta-analysis (*n* = 373).^10,27^ There was very low certainty evidence of no correlation between fear avoidance beliefs and CPM response (*r* = −0.10 [95% CI −0.30 to 0.10], I^2^ = 70%) (Figure 3).

## Discussion

This systematic review has assessed the association between psychological factors, depression, fear avoidance, anxiety and pain catashrophising on CPM response in people with chronic spinal pain. Although most of the investigated psychological factors showed no relationships with CPM response, higher pain catastrophising was correlated with CPM ‘non-responder’ and ‘facilitator’ status in six ‘very-low’ quality studies. Since they partially share overlapping neurobiological mechanisms (i.e. involving some of the same endogenous pathways activation and neurotransmitters), a relationship between the control of negative emotions and regulation of modulatory pathways may have important clinical implications.

### Relationship between psychological factors and CPM response

There is evidence supporting a CPM ‘non-responder’ or ‘facilitator’ status (indicating an impaired regulation of endogenous modulatory pathways) in people with cLBP,^28^ non-traumatic neck pain^29^ and also other clinical conditions, including hip pain,^30^ carpal tunnel syndrome,^31^ patellofemoral pain^32^ and musculoskeletal shoulder pain.^33^ Pain processing and its perception, are influenced partly by endogenous descending controls and factors, including pain catastrophising.^34^ From a perspective of considering both psychological and biological domains, the association between pain catastrophising and CPM response is in accordance with the literature investigating the neurobiological mechanisms underpinning the regulation of negative emotions and endogenous modulatory systems.^34^ Neuroimaging studies have reported that catastrophising and (pain) modulatory circuits share similar brain regions.^35,36^ In a study using a pharmacological supplement (i.e. naltrexone), King et al. manipulated the endogenous pathways and confirmed their role in those pain inhibition processes tested in CPM protocols.^37^ However, it was also reported that pain catastrophising acts as a mediator in regulating opioid-dependent pathways leading ‘high-catastrophiser’ individuals to rely less on endogenous inhibitory systems.^37^ Although the results of the present review revealed a significant association for this domain, the differences in the stimulus or conditioning test sites might explain some of the non-significant findings encountered. This was reported in a study investigating Naloxone, which could ‘block’ the CPM effect when heat pain was used as a conditioning stimulus,^38^ but not with the cold pressor test.^39^ The range of mean ages of the included participants across studies (37–60 years) may have influenced the correlation of pooled data since older adults seem to show a lower CPM response compared to younger adults.^40^

Considering the role that both depression, anxiety and impaired descending pain modulation have on the development of persistent pain, the lack of association between depression and anxiety with CPM response was unexpected.^41^ Two studies evaluated depression using the DASS and the BDI^10,25^ and anxiety was measured with the DASS and PASS tools. An explanation might relate to the test location with the CPM protocol because only Rabey et al.^10^ applied the test stimulus on the lower back region. Both studies^10,25^ utilised different thermal modalities for the conditioning stimulus, and the test stimulus location was also applied in different locations. When measuring the pressure pain threshold, there is good to excellent reliability. However, when measuring the point at which the painfulness of stimulation becomes intolerable retest reliability typically ranges from poor to fair.^16^ Similarly, when using contact heat as a stimulus, the individualised temperature of the contact heat pain test demonstrates fair to excellent reliability as an outcome measure, whereas pain ratings for exposure to contact heat tend to range from poor to fair.^16^

### Clinical implications and future direction

For chronic spinal pain patients, a holistic clinical assessment approach should incorporate an evaluation of associated pain catastrophising. While this systematic review reported an association between pain catastrophising and CPM response, a causal relationship could not be verified. However, since an association between reported pain and pain catastrophising could have clinical implications, investigating whether a causal relationship exists presents an important initial step for future studies. Moreover, the application of a CPM paradigm, which allows delineation of functionality in descending control pathways, strengthens the possibility of tying mechanisms underlying reported pain and pain catastrophising. Managing pain catastrophising, for example, with cognitive behavioural therapy,^42^ and demonstrating that such an intervention results in amelioration of pain alongside improved CPM efficiency, would indicate that a reduction in catastrophising could act as a mediator for the regularisation of pain modulation. Clinically, CPM efficiency restoration is possible with both pharmacological and nonpharmacological (conservative rehabilitation) interventions. Therefore, assessing CPM as a possible prognostic factor and/or predictor of response to therapeutic intervention in patients with chronic pain may be valuable to support decision-making for clinicians in practice when identifying individualised or stratified management options.

### Review limitations

Our review is not without limitations. We acknowledge that chronic spinal pain and psychological factors are often complex and multidimensional. The impact of how ethnicity, co-morbidities (such as obesity or smoking) and how medications may influence the CPM mechanisms require further research. Across all included studies in this review, there were 339 female participants (57%), the influence of the menstrual cycle might influence the CPM paradigm cannot be excluded. Furthermore, five studies included in our systematic review had a mean age population of 50 years and below, limiting our results’ external validity to older adult populations. The identified studies were undertaken in secondary or tertiary care centres, which may result in selection bias and limit generalisability to primary care populations. Furthermore, the included studies were written in the English or Italian language or those that could be translated. This may have resulted in a publication bias of our included studies by language.

## Conclusion

Overall, very low certainty evidence suggests that pain catastrophising is associated with CPM response in people with chronic spinal pain. However, findings need to be considered with caution because of the small number of low-quality studies. To partially address some of the identified limitations and obtain robust evidence, standardised protocols for assessing CPM response in sufficiently powered cohorts are warranted.

## Supplemental Material

Supplemental Material - The association between conditioned pain modulation and psychological factors in people with chronic spinal pain: A systematic review Supplemental Material for The association between conditioned pain modulation and psychological factors in people with chronic spinal pain: A systematic review by Michael Mansfield, Gianluca Roviello, Mick Thacker, Matthew Willett, Kirsty Bannister and Toby Smith in British Journal of Pain
