# Supplementary material for: The association between conditioned pain modulation and psychological factors in people with chronic spinal pain: A systematic review
Source: Br J Pain. 2024 Jan 24;18(4):314–24. doi: 10.1177/20494637241229970 (PMC11289901; doi:10.1177/20494637241229970)
Supplement: Supplemental Material - The association between conditioned pain modulation and psychological factors in people with chronic spinal pain: A systematic review [file sj-pdf-1-bjp-10.1177_20494637241229970.pdf]

Supplementary file 1. Search terms and strategy

Search strategies

**PubMed**	(“conditioned pain” OR “counter-irritation” OR “pain modulation” OR CPM OR "endogenous pain modulation" OR "diffuse noxious inhibitory control" OR DNIC OR “heterotopic noxious conditioning stimulations” OR “endogenous analgesia” OR “pain inhibition” OR "descending pain modulation" OR "descending inhibitory system") AND (“psychological” OR “personality” OR “fear of movement” OR “pain-related fear” OR “pain related fear” OR “fear avoidance” OR “fear-avoidance” OR “kinesiophobia” OR “catastrophizing” OR “catastrophization”[MeSH] OR “fear of pain” OR “fear of injury” OR “self-efficacy” OR “self efficacy” OR “self efficacy”[MeSH] OR “anxiety” OR “anxiety”[MeSH] OR “depression” OR “depression”[MeSH] OR “depressed”)
**MEDLINE** and **EMBASE** (OVID interface)	1. ('conditioned pain' or 'counter-irritation' or 'pain modulation' or CPM or 'endogenous pain modulation' or 'diffuse noxious inhibitory control' or DNIC or 'heterotopic noxious conditioning stimulations' or 'endogenous analgesia' or 'pain inhibition' or 'descending pain modulation' or 'descending inhibitory system').mp.<br>2. ('psychological' or 'personality' or 'fear of movement' or 'pain-related fear' or 'pain related fear' or 'fear avoidance' or 'fear-avoidance' or 'kinesiophobia' or 'catastrophizing' or 'fear of pain' or 'fear of injury' or

	'self-efficacy' or 'self efficacy' or 'anxiety' or 'depression' or 'depressed').mp.<br>3. catastrophization/ or self efficacy/ or anxiety/ or depression/<br>4. 2 or 3<br>5. 1 and 4
**CINHAL** (EBSCO interface)	TXT = (“conditioned pain” OR “counter-irritation” OR “pain modulation” OR CPM OR "endogenous pain modulation" OR "diffuse noxious inhibitory control" OR DNIC OR “heterotopic noxious conditioning stimulations” OR “endogenous analgesia” OR “pain inhibition” OR "descending pain modulation" OR "descending inhibitory system")<br>AND<br>TXT = (“psychological” OR “personality” OR “fear of movement” OR “pain-related fear” OR “pain related fear” OR “fear avoidance” OR “fear-avoidance” OR “kinesiophobia” OR “catastrophizing” OR “fear of pain” OR “fear of injury” OR “self-efficacy” OR “self efficacy” OR “anxiety” OR “depression” OR “depressed”)
